# *Corydalis decumbens* Alleviates the Migration, Phagocytosis, and Inflammatory Response of Macrophages

**DOI:** 10.1155/2023/7000477

**Published:** 2023-02-22

**Authors:** Shanmin Huang, Xuehua Zheng, Weiguo Dong, Yao Lin, Shu Jiang, Haiyin Zheng, Changhui Qian

**Affiliations:** College of Integrated Traditional Chinese and Western Medicine, Fujian University of Traditional Chinese Medicine, Fuzhou 350122, China

## Abstract

**Background:**

The role of *Corydalis decumbens* (CD) in macrophage activation remains unclear, particularly in the Ras homolog family member A (RhoA) signaling pathway. Therefore, the present study aimed to investigate the effect of CD on the viability, proliferation, morphological changes, migration, phagocytosis, differentiation, and release of inflammatory factors and signaling pathways in lipopolysaccharide (LPS)-stimulated RAW264.7 macrophages.

**Methods:**

Cell counting kit-8 and water-soluble tetrazolium salt assays were used to evaluate the viability and proliferation of RAW264.7 macrophages. A transwell assay was examined to assess cell migration. The ingestion of lumisphere assay was employed to detect the phagocytic capacity of macrophages. Phalloidin staining was performed to observe morphological changes in the macrophages. An enzyme-linked immunosorbent assay was performed to quantify inflammation-related cytokines in cell culture supernatants. Cellular immunofluorescence and western blotting were adopted to show the expression of inflammation-related factors, biomarkers of M1/M2 subset macrophages, and factors of the RhoA signaling pathway.

**Results:**

We found that CD increased the viability and proliferation of RAW264.7 macrophages. CD also impaired the migration and phagocytic capacity of macrophages, induced anti-inflammatory M2 macrophage polarization, such as M2-like morphological changes, and upregulated M2 macrophage biomarkers and anti-inflammatory factors. We also observed that CD inactivated the RhoA signaling pathway.

**Conclusions:**

CD mediates the activation of LPS-stimulated macrophages, alleviates the inflammatory responses of macrophages, and activates related signaling pathways induced by LPS.

## 1. Introduction

*Corydalis decumbens* (CD) is a traditional Chinese medicine included in the 2020 edition of the Chinese Pharmacopoeia. It possesses analgesic effects and has shown promise as a potential clinical treatment for sciatica; however, the underlying mechanism remains unclear. Recently, we reported that CD could have high efficacy as an anti-inflammatory, detumescent, and analgesic agent in rat models with sciatica [1]. Furthermore, CD reduced macrophage infiltration and downregulated the expression of proinflammatory cytokines in the microenvironment of damaged sciatic nerves. Based on these results, we investigated the mechanism of CD's functional regulation of macrophages.

Macrophages are important immunological cells crucial for maintaining and restoring homeostatic physiological conditions and play a major role in inflammation in many neurological diseases, including sciatica [2–5]. Following peripheral nerve damage, macrophages infiltrate into the injured nerve microenvironment and play key roles in initiating phagocytic activities and facilitating inflammatory responses to enhance peripheral sensitization. This increases central sensitization and causes persistent pain. Upon polarization state, macrophages are classified as inactivated macrophages (M0 macrophages), classically activated macrophages (M1 macrophages), and alternatively activated macrophages (M2 macrophages) [6, 7]. Activated M1 macrophages may aggravate inflammatory responses, and a blockage of M1 cells can alleviate neuroinflammation. In contrast, M2 macrophages are responsible for eliminating inflammation and initiating tissue repair [8, 9]. In this study, we chose lipopolysaccharide (LPS)-stimulated RAW264.7 macrophages and explored cellular physiological characteristics, such as cell proliferation, polarization, migration, phagocytosis, and cytokine secretion, to analyze the anti-inflammatory mechanism of CD. In addition, we investigated the signaling pathways involved in macrophage activation. Existing data have illustrated that macrophage activation is closely related to the Ras homolog family member A (RhoA) signaling pathway [10–12]. Activated macrophages require dynamic reorganization of cytoskeleton components through RhoA regulation to exert multiple functions, such as phagocytosis, migration, and cell-to-cell interactions. A decrease in RhoA signaling pathway activity in macrophages could inhibit M1 polarization and reduce inflammatory responses [13, 14]. To test whether CD plays a role in the RhoA signaling pathway, which remains unclear, we investigated the expression of RhoA and its effector, Rho-associated protein kinase (ROCK), in macrophages. Overall, our aim was to explore the effects of CD on the activity of LPS-stimulated macrophages to attenuate inflammatory responses and the signaling pathway involved.

## 2. Materials and Methods

### 2.1. Macrophage Culture

RAW264.7 macrophages (Procell Life, China) were cultured in Dulbecco's Modified Eagle Medium/Nutrient Mixture F-12 (DMEM/F12) (Thermo Fisher, USA), which contained 10% fetal bovine serum (FBS) (ExCell, China) and antibiotics (100 U/mL penicillin and 100 mg/mL streptomycin, Thermo Fisher, USA) at 37°C and under 5% CO_2_. To establish the inflammation model, 1 *μ*g/mL LPS (Solarbio, China) was added to the cultured cells and incubated for 24 h. CD injection (Herbi-Sky, China) was then added for 4 h. In this study, the cells were divided into four groups: control (no LPS and CD added), LPS (LPS added), LPS + CD (LPS and CD added), and CD (no LPS added).

### 2.2. Antibodies

The antibodies used were as follows: rabbit anti-RhoA (1 : 300, Abcam, ab187027), rabbit anti-ROCK (1 : 200, Abcam, ab125025), rabbit anti-CD 68 (1 : 200, Abcam, ab125212), rabbit anti-CD80 (1 : 200, Abcam, ab215166), rabbit anti-CD163 (1 : 200, Abcam, ab182422), rabbit anti-CD206 (1 : 200, Abcam, ab64693), rabbit anti-inducible nitric oxide synthase (iNOS, 1 : 200, Abcam, ab178945), mouse antitumor necrosis factor-*α* (TNF-*α*, 1 : 200, Santa Cruz, sc-52746), mouse anti-interleukin-6 (IL-6, 1 : 300, Abcam, ab9324), and mouse anti-IL-10 (1 : 200, Santa Cruz, sc-365858).

### 2.3. Cell Viability Assay

Cell viability was examined using the Cell Counting Kit-8 (CCK-8) assay (MCE, USA). Cells were firstly seeded in a 24-well plate at a density of 1 × 10^5^ cells/well in DMEM/F12 medium. Furthermore, 1 *μ*g/mL LPS was added to the LPS group and the LPS + CD group, respectively. After incubating for 24 h at 37°C, the cells were resuspended and seeded in a 96-well plate at a density of 1 × 10^4^ cells/well. CD was then added to the LPS + CD and CD groups and then incubated for 4 h. Thereafter, 10 *μ*l CCK-8 solution was added to each well, and the cells were incubated for 1 h at 37°C. The absorbance at 450 nm was shown using a microplate reader (Tecan, Switzerland).

### 2.4. Cell Proliferation Assay

Cell proliferation was detected using a water-soluble tetrazolium salt (WST) assay (Abcam, UK). Macrophages were cultured in the same conditions as above. 10 *μ*L WST reagent was added to 0.1 mL of medium in each well of 96-well plates and incubated for 1 h at 37°C. The absorbance at 450 nm was measured using a microplate reader.

### 2.5. Migration Assay

Macrophage migration was evaluated with a transwell cell migration assay as previously described [15]. Briefly, the chambers with 8 *μ*m pores (Corning Costar, USA) were firstly pretreated with DMEM/F12 medium. Then, 100 *μ*L of DMEM/F12 containing 1 × 10^5^ macrophages and 1% FBS was added into the upper chamber, and the lower compartment was filled with 600 *μ*L medium containing 10% FBS without cells. After incubation for 18 h, the transwell membrane was fixed with 4% paraformaldehyde (PFA) (Macklin, China) for 30 min. After removing the nonmigrated cells from the upper chamber with a cotton swab, the migrated cells were stained using a 0.1% crystal violet solution for 30 min. For quantification, 6 pictures of each membrane were photographed with an inverted microscope (Leica, Germany).

### 2.6. Phagocytic Capability Assay

The ingestion of lumisphere was used to assess phagocytosis in macrophages [16]. Macrophages were grown on 24-well chambered slides at a density of 1 × 10^5^ cells/well. These cells were incubated for approximately 16 hours, and then 0.1 mg/mL fluorescent green microspheres (1 *μ*m in diameter, BaseLine, China) were added to the culture medium for 3 h. Cells were then fixed with 4% PFA. The number of lumisphere endocytosed by each cell was calculated (20 cells per group were evaluated) by fluorescent microscopy (Nikon, Japan).

### 2.7. Phalloidin Staining

To facilitate phalloidin staining, 1 × 105 cells were fixed with 4% PFA for 20 min, permeabilized with 1% Triton X-100 for 1 h, and then blocked with 5% bovine serum albumin (BSA) (Absin, China) in phosphate buffer saline (PBS) for 1 h before applying fluorescein isothiocyanate (FITC)-phalloidin (Sigma, USA) for 1 h. Specimens were mounted in Vectashield medium (Vector, USA). All pictures were captured by fluorescent microscopy.

### 2.8. Immunofluorescence

In this study, 1 × 10^5^ cells were firstly fixed by immersion in 4% PFA for 20 min and then infiltrated in 1% Triton X-100 for 1 h and incubated with 5% BSA in PBS for 30 min. The cells were then treated with primary antibodies for at least 24 h at 4°C, followed by fluorescent-conjugated secondary antibodies for 2 h. Finally, the slides were mounted using Vectashield medium. The stained cells were observed by fluorescent microscopy, and immunostained pictures were photographed using a high-resolution digital camera.

### 2.9. Western Blotting

Cells were harvested, and the proteins were extracted from collected cells using RIPA lysis buffer (CST, USA), run on 10%–12% SDS-PAGE gels, and then transferred from the gels to polyvinylidene difluoride membrane (PVDF) (Millipore, USA). The membrane was blocked in 5% BSA for 2 h, stained with the primary antibody for 24 h at 4°C, and then blots were probed with secondary antibodies for 2 h. Enhanced chemiluminescence was visualized on the PVDF membrane, and the band density was quantified using Image-Pro Plus 6.0.

### 2.10. Enzyme-Linked Immunosorbent Assay

The supernatants of cultured macrophages were assayed using an enzyme-linked immunosorbent assay (ELISA) (Boster, USA) array following the manufacturer's protocol to measure the expression of proinflammatory cytokines TNF-*α* and IL-6 and anti-inflammatory factor IL-10. The culture supernatants were collected and added to each ELISA plate. The absorbance at 450 nm was measured by a microplate reader.

### 2.11. Image Analysis

For quantification of fluorescence intensity, the images in 3 sections per group were taken at 40x magnification in areas, and 6 random fields per section were considered. The result was quantified with Image-Pro Plus 6.0.

### 2.12. Statistical Analysis

SPSS 23 software (IBM, USA) was used for the statistical analyses. Each independent experiment was performed in triplicate, and the results were presented as the mean ± standard error. The difference between groups was analyzed by one-way analysis of variance, or a student's *t*-test. The statistical significance was set at *P* < 0.05.

## 3. Results

### 3.1. CD Upregulates the Viability and Proliferation of LPS-Stimulated Macrophages

**Figure 1:**
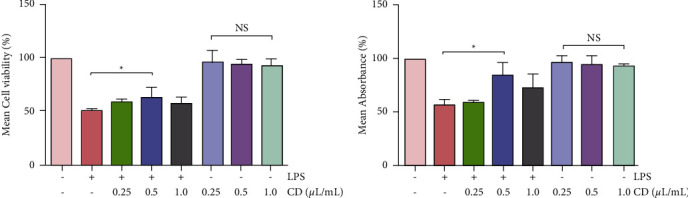
CD increased the viability and proliferation of LPS-stimulated macrophages (*n* = 3). (a) Statistical diagram showing macrophage viability using the CCK-8 assay. (b) Statistical diagram showing the absorbance of the macrophages using WST assays. ^*∗*^*P* < 0.05, ^*NS*^*P* > 0.05.

Quantitative analysis of the cultured cells was performed using CCK-8 and WST assays. We observed the effect at three different CD concentrations (0.25, 0.5, and 1 *μ*L/mL), with and without LPS stimulation, and summarized the relative cell viability and proliferation after the incubation (100% for untreated cells at 24 h). The data showed that the viability of macrophages treated with the three concentrations of CD without LPS stimulation did not change (Figure 1). These results suggest that CD has no cytotoxic effect on RAW264.7 macrophages. In addition, LPS-stimulated cells exhibited significantly lower viability and proliferation than those in the other groups. When CD was added, cell viability and proliferation increased, particularly in the 0.5 *μ*L/mL group. As a result, 0.5 *μ*L/mL CD was available during the experimental period.

### 3.2. CD Suppresses the Migration and Phagocytic Capacity of Macrophages

**Figure 2:**
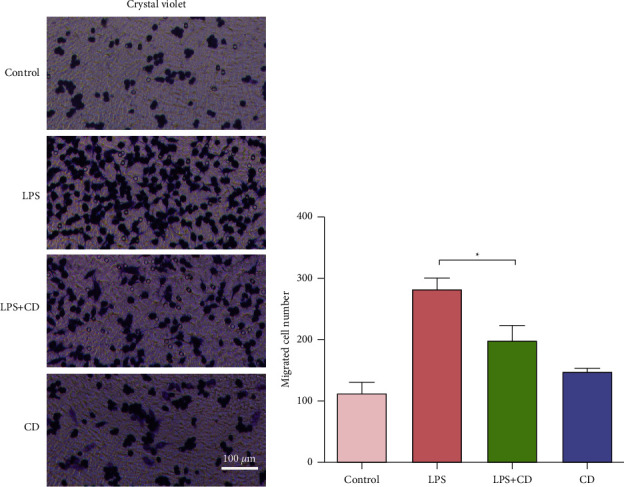
CD suppresses the migration of LPS-stimulated macrophages (*n* = 3). (a) Representative images showing macrophage migration in each group. (b) Quantification of migrated macrophages showing reduced migration after CD treatment. ^*∗*^*P* < 0.05.

In this study, we focused on the comparison of the cultured macrophages between the LPS and LPS + CD groups. Firstly, we visualized the migrated cells in the transwell lower chamber to compare the chemotactic ability of macrophages. We found that macrophage migration was significantly enhanced after LPS stimulation in the LPS group. Compared to the macrophages in the LPS group, the cells showed a slower migration rate in the LPS + CD group (Figure 2).

**Figure 3:**
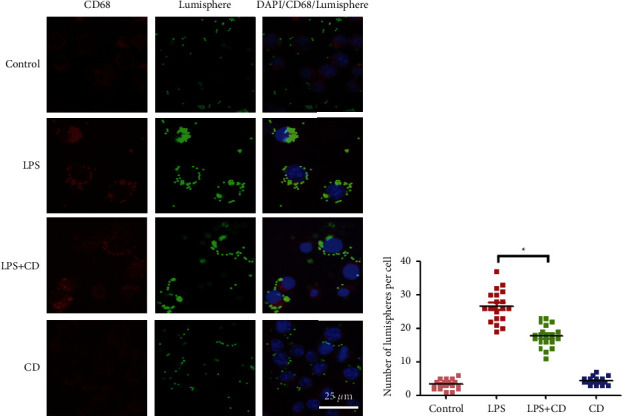
CD suppresses macrophage phagocytosis (*n* = 3). (a) Double immunofluorescence staining of CD68 stained macrophages and FITC-labeled lumisphere. (b) Statistical diagram showing the number of internalized lumisphere in each macrophage that were downregulated upon CD treatment. ^*∗*^*P* < 0.05.

Next, we examined whether CD altered the phagocytic capability of macrophages. Macrophages were labeled with CD68 (a macrophage marker) and 0.1 mg/mL FITC-conjugated lumisphere. After LPS treatment, the number of macrophages that were fluorescence-labeled by lumisphere ingestion increased, but there was a greater decrease in internalized lumisphere within each macrophage in the LPS + CD group than in the LPS group (Figure 3).

### 3.3. CD Induces Anti-Inflammatory M2 Macrophage Polarization

**Figure 4:**
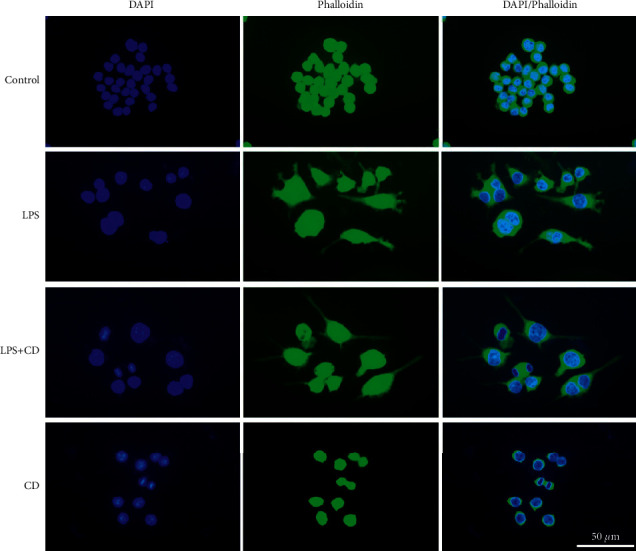
Morphological characteristics of macrophages in different groups (*n* = 3). Lamellipodia-like pseudopodia protrusions showing macrophages in the LPS group. The cell morphology was spread well enough to show elongation to form M2-like macrophages upon CD treatment.

To study macrophage polarization, we observed changes in cellular morphology. Mounting evidence [16, 17] suggests M1-polarized macrophages were typically flattened with lamellipodia-like protrusions, while M2-polarized macrophages were elongated into spindle-like shapes. In this study, macrophage morphology was examined by FITC-phalloidin staining. We observed that the cells exhibited different shapes (Figure 4). In the untreated control and CD groups, the macrophages were generally round. Compared to the cultured macrophage in the LPS group, there were fewer cells that formed lamellipodia-like structures and more cells that elongated their shape in the LPS + CD group.

**Figure 5:**
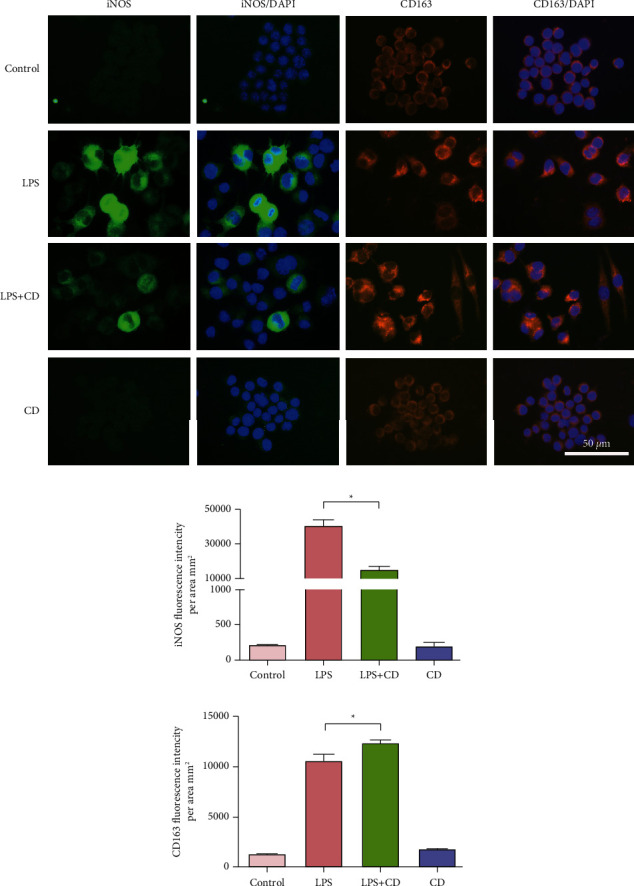
Analyses of expression of M1/M2 markers in different groups (*n* = 3). (a) The representative images show M1 marker (iNOS) expression and M2 marker (CD163) in cultured macrophages of different groups. (b) and (c) Statistical diagrams showing down-regulated iNOS expression and upregulated CD163 expression upon CD treatment. ^*∗*^*P* < 0.05.

**Figure 6:**
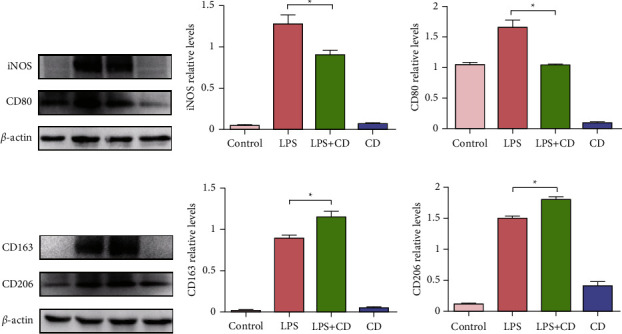
Western blotting analysis of M1/M2 markers in macrophages (*n* = 3). (a–c) Western blotting analysis indicated that iNOS and CD80 levels declined upon CD treatment. (d–f) CD163 and CD206 levels increased upon CDP treatment. ^*∗*^*P* < 0.05.

Additionally, M1/M2 macrophage polarization changes were compared to explain macrophage responses during inflammation caused by LPS. Immunofluorescence staining showed that the M1 population biomarker, iNOS, increased in the group with LPS-stimulated macrophages but not in the LPS + CD group. The LPS-stimulated cells had a larger population of M1 macrophages than CD-treated cells. Meanwhile, the M2 population marker, CD163, was increased in the macrophages of the LPS + CD group but not in those of the LPS group (Figure 5). To further illustrate the macrophage subset population, western blotting was performed. The results showed significant upregulation of M1 biomarkers (iNOS and CD80) and downregulation of M2 biomarkers (CD163 and CD206) (Figure 6). These findings suggest that CD treatment of macrophages may increase macrophage polarization towards the M2 phenotype.

**Figure 7:**
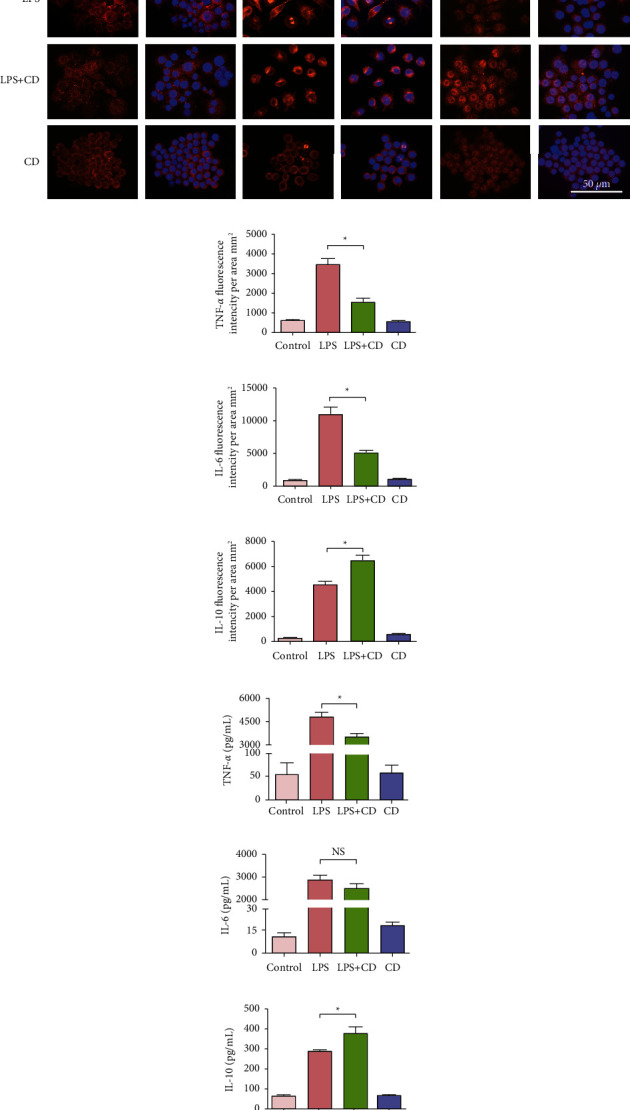
Analyses of the production of inflammatory cytokines in different groups (*n* = 3). (a) The representative images show proinflammatory factors TNF-*α* and IL-6 and anti-inflammatory cytokine IL-10 expression in cultured macrophages in different groups. (b–d) Statistical diagrams showing downregulated TNF-*α* and IL-6 levels and upregulated IL-10 levels in macrophages. (e–g) Statistical diagram showing downregulated TNF-*α* and IL-6 levels and upregulated IL-10 levels in the supernatant of macrophage culture via ELISA assays. ^*∗*^*P* < 0.05, ^*NS*^*P* > 0.05.

Furthermore, we considered the production of inflammatory factors, TNF-*α*, IL-6, and IL-10. Expression of the proinflammatory factors, TNF-*α* and IL-6, enhanced significantly in the macrophage after LPS stimulation. However, the expression of proinflammatory factors in macrophages in the LPS + CD group was considerably lower than that in the LPS group (Figures 7(a)–7(d)). Meanwhile, CD-induced anti-inflammatory factor IL-10 expression increased significantly in macrophages of the LPS + CD group compared to that in the LPS group. Finally, we studied the production of inflammatory cytokines using ELISA. Although not all differences were statistically significant, the trend in proinflammatory or anti-inflammatory factor secretion was consistent with the fluorescence results (Figures 7(e)–7(g)). These results suggest that the downregulation of proinflammatory factors TNF-*α* and IL-6 and upregulation in anti-inflammatory cytokine IL-10 expression in macrophages was triggered by CD treatment in the LPS + CD group.

### 3.4. CD Inactivates the RhoA Signaling Pathway

**Figure 8:**
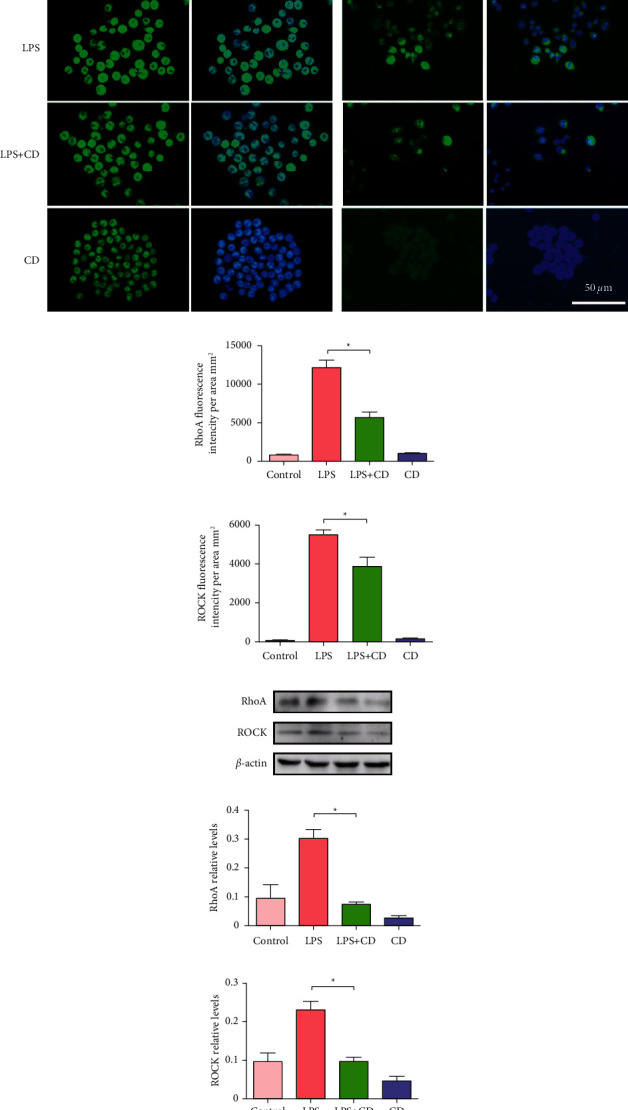
Analyses of expression of RhoA and ROCK in different groups (*n* = 3). (a) The representative images show RhoA and ROCK expressions in cultured macrophages in the different groups. (b, c) Statistical diagrams showing downregulated RhoA or ROCK expression upon CD treatment. (d–f) Western blotting analysis indicated RhoA and ROCK levels declined upon CD treatment. ^*∗*^*P* < 0.05.

To evaluate whether the RhoA signaling pathway is responsible for mediating CD treatment of LPS-stimulated macrophages, RhoA and ROCK were detected. Compared to the LPS group, expressions of RhoA and ROCK were downregulated in the presence of CD in the LPS + CD group (Figures 8(a)–8(c)). Western blotting verified that expressions of RhoA and ROCK were remarkably decreased in the LPS + CD group (Figures 8(d)–8(f)). These results led us to hypothesize that the RhoA pathway may be associated with the suppression of CD by LPS stimulation in macrophages.

## 4. Discussion

An increasing number of reports have shown that the LPS-stimulated RAW264.7 macrophage model is highly adaptable to inflammatory response research [18–20]. In vitro stimulation with LPS can drive the polarization of M1 macrophages, regardless of morphology or function [21, 22]. Herein, we developed an in vitro study and found that LPS stimulation induced the upregulation of phagocytosis, migration, and expression of proinflammatory factors, as well as the downregulation of proliferation and anti-inflammatory factor expression in cultured macrophages. These results agree with those of previous reports [23–25].

In this study, we found that CD increased the viability and proliferation of RAW264.7 macrophages (Figure 1) and suppressed macrophage migration and phagocytosis (Figures 2 and 3). These findings reveal that CD partially influences LPS stimulation of macrophages by alleviating macrophage migration and phagocytosis, the two important biological processes during inflammatory responses [26, 27].

As is well known, macrophages can trigger inflammatory responses and play a crucial role in altering proinflammatory or anti-inflammatory microenvironments [28–30]. Phenotypic switching plays a key role in eliminating inflammation. In this study, we observed that CD treatment changed the shape of macrophages, elongating them to form M2-like macrophages (Figure 4). Generally, the M1 subtype macrophage produces abundant proinflammatory cytokines that hinder tissue repair. Compared to the proinflammatory M1 macrophages, the M2 type is capable of secreting anti-inflammatory factors and reducing inflammatory responses [31, 32]. Our study found that CD enhanced the expression of M2 biomarkers in LPS-stimulated macrophages, such as CD163 and CD206 (Figures 5 and 6). Thus, we postulated that CD effects on LPS-stimulated macrophages do not induce strong inflammation but are helpful for the recovery of injured cells through M2 polarization. Furthermore, the upregulated secretion of the anti-inflammatory factor, IL-10, and downregulated production of proinflammatory cytokines, TNF-*α* and IL-6, in the culture supernatants strongly implied that CD-induced M2 phenotype polarization, forcefully shifted the macrophages from M1 to M2 subset, and mediated the anti-inflammatory effects via the regulation of cytokines (Figure 7).

The RhoA signaling pathway is closely related to the activation of macrophage development and inflammatory responses. Regulation of the cytoskeleton through the RhoA pathway has been demonstrated to alter the shape and function of macrophages [33, 34]. Choi et al. [13] found that Rhosin, a RhoA inhibitor, can prevent RhoA interaction with its GEFs and decrease M1 macrophage polarization and inflammatory responses by modulating dynamic changes in the actin cytoskeleton. Inactivation of RhoA GTPase can initiate multiple consequences on host cells, such as suppression of cell migration or cell death [35, 36]. Our study found that RhoA and ROCK expression were downregulated upon CD treatment (Figure 8). This result suggests that RhoA control may affect macrophage subset polarization and function by causing morphological and functional changes in macrophages, although the detailed mechanisms are yet to be elucidated.

Taken together, the novel findings of this study were as follows: CD (1) increased the proliferation and viability of macrophages, (2) polarized macrophages towards M2 cells, (3) inhibited migration and phagocytic capacity, (4) contributed to M2 chemokine marker expression in macrophages, and (5) decreased pro-inflammatory factor expression while increasing anti-inflammatory factor expression.

## 5. Conclusions

Our findings highlight that CD can regulate the activation of LPS-stimulated macrophages and exert anti-inflammatory effects. In addition, the RhoA signaling pathway is vital in CD treatment, and more intensive studies are needed for further research.

## Data Availability

The data presented in current study are available within this article.
